# Intraspecific and interspecific competition induces density‐dependent habitat niche shifts in an endangered steppe bird

**DOI:** 10.1002/ece3.3444

**Published:** 2017-10-17

**Authors:** Rocío Tarjuelo, Manuel B. Morales, Beatriz Arroyo, Santiago Mañosa, Gerard Bota, Fabián Casas, Juan Traba

**Affiliations:** ^1^ Terrestrial Ecology Group (TEG) Department of Ecology Universidad Autónoma de Madrid Madrid Spain; ^2^ Universidad Rey Juan Carlos Móstoles Spain; ^3^ Instituto de Investigación en Recursos Cinegéticos, IREC (CSIC, UCLM, JCCM) Ciudad Real Spain; ^4^ Department de Biologia Evolutiva, Ecologia i Ciències Ambientals Institut de Recerca de la Biodiversitat (IRBio) Facultat de Biologia Universitat de Barcelona Barcelona Catalonia Spain; ^5^ Biodiversity and Animal Conservation Lab Forest Science Center of Catalonia (CTFC) Solsona Catalonia Spain; ^6^ Estación Experimental de Zonas Áridas (EEZA‐CSIC) Almería Spain; ^7^ Department of Biology University of Maryland College Park MD USA

**Keywords:** ecological release, kernel density estimators, *Otis tarda*, species coexistence, *Tetrax tetrax*

## Abstract

Interspecific competition is a dominant force in animal communities that induces niche shifts in ecological and evolutionary time. If competition occurs, niche expansion can be expected when the competitor disappears because resources previously inaccessible due to competitive constraints can then be exploited (i.e., ecological release). Here, we aimed to determine the potential effects of interspecific competition between the little bustard (*Tetrax tetrax*) and the great bustard (*Otis tarda*) using a multidimensional niche approach with habitat distribution data. We explored whether the degree of niche overlap between the species was a density‐dependent function of interspecific competition. We then looked for evidences of ecological release by comparing measures of niche breadth and position of the little bustard between allopatric and sympatric situations. Furthermore, we evaluated whether niche shifts could depend not only on the presence of great bustard but also on the density of little and great bustards. The habitat niches of these bustard species partially overlapped when co‐occurring, but we found no relationship between degree of overlap and great bustard density. In the presence of the competitor, little bustard's niche was displaced toward increased use of the species' primary habitat. Little bustard's niche breadth decreased proportionally with great bustard density in sympatric sites, in consistence with theory. Overall, our results suggest that density‐dependent variation in little bustard's niche is the outcome of interspecific competition with the great bustard. The use of computational tools like kernel density estimators to obtain multidimensional niches should bring novel insights on how species' ecological niches behave under the effects of interspecific competition in ecological communities.

## 1 INTRODUCTION

The role of interspecific competition in structuring ecological communities and evolutionary diversification is a crucial long‐standing debate among ecologists, which can be addressed within the theoretical framework of ecological niche (Bolnick et al., 2010; Case & Gilpin, 1974; Chase & Leibold, 2003; Chesson, 1991). Competition theory postulates that species must differ in their ecological niches in order to attain a stable coexistence (Chesson, 1991; Leibold, 1995). Otherwise, ecologically similar species that share a limiting resource engage in competition and the species with superior abilities eventually exclude the inferior competitor (Gause, 1934; Human & Gordon, 1996). One of the most prominent ecological mechanisms by which coexisting species resolve their competition is habitat partitioning (Morris, 2003; Rosenzweig, 1981). Interspecific competition is attenuated by a differential habitat use that segregates the species' habitat niches. The habitat niche can be understood as a multidimensional hypervolume (sensu Hutchinson, 1957), where each dimension corresponds to a different habitat exploited as a resource by the species (Chase & Leibold, 2003; Schoener, 1989). By integrating ecological niche and habitat selection theories with the study of habitat niche variation of putative competitors, we can gain novel insights on competition theory.

Most theoretical models of habitat selection assume that coexisting species spatially segregate in different habitats in order to avoid the negative cost of interspecific competition (Morris, 1988; Rosenzweig, 1981). This means that species' habitat niches should not overlap when competing species coexist in a stable manner. Certainly, low niche overlap has been documented between coexisting species currently competing (Schoener, 1982; Smith, Grant, Grant, Abbott, & Abbott, 1978). However, low values of niche overlap may also indicate evolutionary divergence in the species' habitat preferences due to past competition (Connell, 1980). In this case, interspecific competition no longer shapes the habitat distribution of coexisting species, which obeys only to a differential habitat selection. Therefore, the degree of niche overlap does not by itself allow to disentangle whether interspecific competition is currently operating between coexisting species and additional evidences of niche variation are required.

If competition occurs, niche expansion can also be expected when the competitor disappears (i.e., ecological release) because resources previously inaccessible due to competitive constraints can then be exploited (Bolnick et al., 2010; Schoener, 1989). The use of a wider range of habitats in allopatric situations expands the species habitat niche, which then approaches the species' fundamental niche (Morris, 1988). In addition, changes in the habitat distribution due to ecological release may be noticed by displacements of niche position (Adams, 2004), which is often described as the optimum or average value of the species niche (Barnagaud et al., 2012; Williams, Araújo, & Rasmont, 2007), under allopatric and sympatric conditions.

However, it has been theoretically demonstrated that competing species do not necessarily segregate in different habitats when co‐occurring (e.g., Morris, 1999, 2009). Rather, the habitat selection pattern balances intra‐ and interspecific competitive costs on fitness, so competing species can simultaneously use a shared habitat depending upon both species density. Therefore, ecological release from interspecific competition should be a density‐dependent process in which niche shifts depend on the intensity of competition (Pianka, 1974; Young, 2004). Certainly, niche segregation is not necessarily absolute and a permissible degree of niche overlap is more likely to occur in nature (May & Mac Arthur, 1972). In accordance with the “niche overlap hypothesis”, this tolerable upper limit of niche overlap between competing species varies inversely with the intensity of interspecific competition (Pianka, 1974). Habitat niche breadth should decrease with increased density of the competitor due to lower proportional use of the shared habitat (Morris, 2009). Intraspecific competition, however, has opposite effects on a species' niche because organisms diversify resource use to reduce competitive costs (Svanbäck & Bolnick, 2007). Therefore, habitat niche breadth should proportionally increase with the density of conspecifics. Similarly, displacements of niche position should mimic density‐dependent adjustments of habitat distribution caused by inter‐ and intraspecific competition. Despite the relevant role of habitat selection in regulating community structure (Morris, 1988), little is known about the density‐dependent effects of competition on the species' habitat niche variations and empirical evidence is very scarce (Benítez‐López, Viñuela, Suárez, Hervás, & García, 2014; Young, 2004).

This study focused on two sympatric steppe birds to investigate how competition may influence variation in habitat niches. Our model species is the little bustard (*Tetrax tetrax*), a medium‐sized steppe bird which inhabits cereal farmlands in western Europe (Cramp & Simmons, 1980). This species may competitively interact with the great bustard (*Otis tarda*), an ecologically and phylogenetically close species (Broders, Osborne, & Wink, 2003), which frequently co‐occur in many regions across their distribution. The little and great bustard are endangered species currently classified as “near threatened” and “vulnerable,” respectively (IUCN, 2017). During the breeding season, these bustard species show certain similarities in their habitat use and spatial distribution patterns that may cause competition at high densities (Tarjuelo, Traba, Morales, & Morris, 2017). The little bustard is an exploded lek species in which males establish loosely aggregated territories (Jiguet, Arroyo, & Bretagnolle, 2000), preferentially in semi‐permanent agrarian habitats like short‐ and long‐term fallows as well as legume crops (Delgado, Traba, García de la Morena, & Morales, 2010; Morales, García, & Arroyo, 2005; Wolff, Paul, Martin, & Bretagnolle, 2001). The great bustard does not show marked preferences among the main agrarian habitats (Morales, Suárez, & García de la Morena, 2006). This species uses habitats depending upon their relative availability, and conspecific attraction is a major force determining its distribution (Alonso et al., 2004; Lane, Alonso, & Martín, 2001; López‐Jamar, Casas, Díaz, & Morales, 2011; Tarjuelo, Morales, Traba, & Delgado, 2014). Competition between both bustard species is asymmetric and occurs in cereals, the most abundant habitat in these agricultural landscapes and secondarily used by the little bustard (Tarjuelo et al., 2017). The great bustard behaves as the dominant competitor by altering the habitat use of the little bustard, which is gradually displaced from cereals toward its primary habitat. This fact may increase levels of intraspecific competition and force some little bustards to move into other secondary and low‐quality habitats (Tarjuelo et al., 2017). Because interspecific competition between these bustard species is not resolved by a complete spatial segregation (both species are often found simultaneously occupying the same habitats), these species may constitute a good system to gain novel insights into ecological niche theory with relevant implications for the conservation of competing populations.

Here, we evaluate the potential effects of intra‐ and interspecific competition between the little and great bustards on little bustard's habitat niche within the framework of ecological niche theory. We use a methodological approach recently applied in this field to calculate multidimensional niches (Blonder, Lamanna, Violle, & Enquist, 2014; Broennimann et al., 2012; Petitpierre et al., 2012). Although community assembly studies often assume that coexisting species segregate along one crucial niche dimension to avoid competitive exclusion (e.g., Kimura & Chiba, 2010; Stuart et al., 2014), it seems more realistic to consider that multiple interacting niche dimensions modulate the process of species coexistence. We explore variation in three components of ecological niche: overlap, breadth, and position. According to the niche release hypothesis (Schoener, 1989), the presence of great bustards should impose competitive restrictions to habitat use by little bustards, particularly by limiting the access to the secondary habitat (cereal), thereby forcing an increased use of primary habitats (Tarjuelo et al., 2017). This should induce a decrease in little bustard's habitat niche breadth and a niche displacement toward increased use of fallows and natural vegetation. We expect these shifts to be density‐dependent because interspecific competition and its effects intensify with great bustard density. As a consequence of gradual niche segregation, we also expect to find a negative relationship between niche overlap and great bustard density (niche overlap hypothesis—Pianka, 1974). On the contrary, intraspecific competition causes diversified resource use and expands a species' niche (Svanbäck & Bolnick, 2007). Therefore, we expect little bustard density to be positively related to little bustard's niche breadth, and a displacement of niche position toward a higher use of cereals as little bustard density increases.

## 2 METHODS

### 2.1 Study sites

This study was conducted in nine different sites across Spain between 2006 and 2012. Seven sites were located in central Spain and two in the northeast (Table 1). All study sites are under Mediterranean climate and dominated by a mosaic landscape of different agrarian substrates typical of extensive cereal farmlands with a 2‐year rotation system. Dry cereals (mainly wheat *Triticum* spp., barley *Hordeum vulgare*, and oats *Avena* spp.) and ploughed fields (plots unsown for the year to allow soil recovery, but where ploughing is regularly used to prevent weed growth) represent the main agrarian substrates (ca. 50% of the surface), followed by fallow fields with vegetation cover of different ages. Legume crops (*Vicia* spp.*, Pisum sativum* or *Lathyrus sativus*) are also cultivated although not in all the study sites or years. Other minor land uses are vineyards *Vitis vinifera*, olive groves *Olea europaea*, almond orchards *Prunus dulcis*, pastures, and urbanized areas. The little bustard was present in all study sites whereas the great bustard was absent in La Solana, Bellmunt, and Belianes. This fact allows for the evaluation of differences in niche breadth and position between allopatric and sympatric situations.

**Table 1:** Study years for each study sites as well as their location within Spain and geographical coordinates. The mean (±*SD*) per site density of little bustards (number of males per km^2^) and great bustards (number of individuals per km^2^) inside each minimum convex polygon (MCP) is provided together with the mean size (±*SD*) of each site MCP (km^2^). Daganzo and Camarma have data from only 1 year, and thus, standard deviation was not calculated

Site	Year	Region	Coordinates	Size of MCP	Little bustard density	Great bustard density
Campo Real	2010–2012	Central	40°19′N, 3°18′W	8.41 ± 0.30	5.60 ± 0.65	7.48 ± 1.22
Valdetorres	2010–2011	Central	40°40′N, 3°25′W	5.73 ± 3.33	2.56 ± 0.99	20.85 ± 10.76
Daganzo	2010	Central	40°34′N, 3°27′W	4.68	2.13	5.98
Camarma	2006	Central	40°32′N, 3°22′W	41.94	0.50	3.39
Calatrava North	2007–2011	Central	38°56′N, 3°53′W	11.19 ± 2.25	3.79 ± 1.23	3.93 ± 4.02
Calatrava South	2007–2011	Central	38°52′N, 3°57′W	11.82 ± 3.24	4.59 ± 0.45	0.38 ± 0.32
La Solana	2010–2011	Central	38°55′N, 3°13′W	14.33 ± 8.58	2.70 ± 0.97	0
Bellmunt	2008–2011	Northeast	41°47′N, 0°57′E	9.58 ± 1.00	7.66 ± 1.96	0
Belianes	2008, 2010–2011	Northeast	41°35′N, 0°59′E	22.19 ± 7.59	6.06 ± 0.97	0

### 2.2 Bustards and habitat data

Little and great bustard censuses were carried out between April and May, which encompasses both species' mating seasons, when birds are conspicuous (Cramp & Simmons, 1980). Surveys were conducted by car along routes using the net of roads and tracks available in each study site. The high density and spatial configuration of roads and tracks ensured accurate censuses of both bustard species (see details in, e.g., Alonso et al., 2004; Morales, Traba, Carriles, Delgado, & García de la Morena, 2008). Stops were routinely made at every 500 m to scan the surroundings using binoculars and spotting scope, mapping all birds detected. Surveys were made during the first three hours after sunrise, and the last 3 hr before sunset when birds are most active (Cramp & Simmons, 1980). The courtship behavior of little bustard males incorporates snort‐calls and jumps accompanied by wing‐flashings, which allow them to be also detected acoustically and accurately located. We did not consider little bustard female observations in the analysis as their secretive behavior hinders their detection and leads to a severe (but unquantifiable and potentially variable among sites) underestimation of their numbers. Great bustards are often found aggregated together in arenas given their lek mating system (Alonso et al., 2004; Morales & Martín, 2002), and the number of individuals of both sexes in each flock was also determined. Because the presence of both displaying males and attending females in great bustard leks, along with nesting great bustard females, may interfere in the establishment of little bustard breeding territories, we considered both male and female great bustards in the analysis.

Habitat availability was estimated from land‐use maps elaborated from field surveys immediately after bird censuses in each study site and year. Each field was assigned to one of the following seven habitat types: (1) cereal; (2) ploughed field; (3) leguminous crop; (4) 1‐year fallow (hereafter young fallow); (5) fallow older than 2 years and short shrubland (hereafter natural vegetation); (6) dry woody culture, including olive groves, vineyards, and almond tree orchards; and (7) others, which encompasses minority substrates avoided by the species like urban areas, or forests.

### 2.3 Measures of habitat niche shifts and niche overlap

We generated the multidimensional niche hyperspace of these bustard species using information on habitat cover. Study sites are often arbitrarily delimitated, and areas falling outside the local distribution of the species may be included within the study site boundaries. This fact can bias measurements of habitat composition or estimates of species density (Aebischer, Robertson, & Kenward, 1993). In order to avoid this, we first delimitated the area used by both species in each study site and year using the minimum convex polygon (MCP) created with all bustard observations. A set of random points equal to the sum of little and great bustard individuals was generated inside each MCP, fixing a minimum number of 30 random points (details on each habitat surface are provided in Appendix S1, Table S1). The number of observations per site and year ranged between 10 and 174 and 0 and 142 for the little and the great bustard, respectively. Habitat composition was then determined inside a buffer of 100 m around each random or bustard observation point and the proportion of each habitat type extracted. We selected a radius of 100 m based on previous knowledge on little bustard home range areas (Delgado et al., 2010). Next, we performed a principal component analysis (PCA) with the habitat variables in order to summarize habitat within and across study sites and to attain ecological gradients that could be interpreted as species' niche dimensions (e.g., Benítez‐López et al., 2014; Morales et al., 2008; Traba, Morales, Carmona, & Delgado, 2015). The PCA was built using the random and bustard points of all study sites and years (see Traba et al., 2015 for a similar approach).

**Figure 1:**
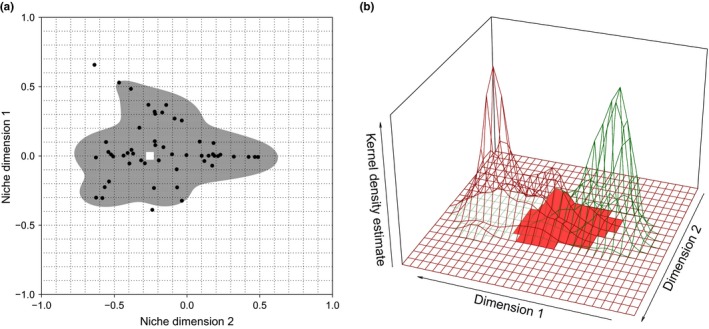
An example of a two‐dimensional kernel density estimator (KDE) procedure used to obtain the species' habitat niches from habitat data. Graph (a) KDEs were calculated from set coordinates in order to obtain comparable values for the analysis (cross points of dotted lines). The gray region reflects the 95% KDE volume of highest probability. This 95% KDE region is used for the analysis in order to avoid the influence of outlier observations. The white square represents niche position, where the KDE attained its highest density value. Niche breadth was estimated as the number of cells falling within the 95% KDE. Black dots are the values of each niche dimension for each bird observation. Graph (b) niche overlap was calculated as the volume under the area where two KDEs intersect. Brown and green lines delimitate two bivariate kernel density functions. The red surface reflects the region where both functions overlap

The species' multidimensional habitat niches were defined using a nonparametric kernel density estimator procedure (KDE; Mouillot et al., 2005). KDEs provide smooth functions that do not assume normal distribution for the niche dimensions and can easily incorporate complex geometries due to their high flexibility (Geange, Pledger, Burns, & Shima, 2011; Mouillot et al., 2005). One KDE was built for each species per study site and year for which enough observations were available (*n* = 26 for little bustards and *n* = 9 for great bustards). We similarly built KDEs only with random points creating “environmental niches” in order to control for the effects of habitat availability on little bustard habitat niche (*n* = 26). We built two‐dimensional KDEs for possible combinations of pairs of PC axes with ecological meaning for the species, instead of the one‐dimensional KDEs used in other studies (e.g., Benítez‐López et al., 2014; Traba et al., 2015). Our approach might better reflect the process of individual habitat choice than single‐variable niche spaces. We fixed a minimum of five bird observations per dimension to estimate KDEs (Mouillot et al., 2005). All KDEs were weighted by the number of individuals in the observation. We used the multivariate plug‐in bandwidth selection with unconstrained matrices (Chacon & Duong, 2010). For the subsequent calculation of niche measurements (overlap, breadth, and position), we considered the region defined by the 95% volume of the KDEs with highest probability (Figure 1a). We left a 5% KDE region outside the niche space in order to avoid the influence of outlier observations.

We calculated niche overlap for nine study sites and years (Campo Real 2010–2012; Valdetorres 2010–2011; Daganzo 2010; Camarma 2006; Calatrava North 2008–2009) where little and great bustard co‐occurred and the number of bird observations allowed for KDE calculation (a minimum of 10 birds observed, five animals per dimension). The calculation of niche overlap required that the two‐dimensional habitat niches of little and great bustard were estimated inside a common niche space and the probability density functions evaluated in the same points in order to be comparable. Therefore, we set the coordinates (at regular intervals) of the two‐dimensional niche in which the probability density functions would be evaluated (Figure 1a). As the volume under the two‐dimensional KDE area sums 1, niche overlap was estimated as the volume under the area where a given pair of little and great bustard KDEs overlap (Stine & Heyse, 2001; Mouillot et al., 2005; Figure 1b). Zero values indicate no overlap whereas values of 1 reflect complete niche overlap.

Niche breadth was calculated as the number of cells of the two‐dimensional KDE falling within the 95% defined region (Figure 1a). Niche position was estimated as the coordinates of each niche dimension where the two‐dimensional kernel density function attained the maximum probability value (Figure 1a). Niche breadth and position for the little bustard and the environmental niche were calculated for all the study sites and years. Again, we set the coordinates of the two PC dimensions where the probability density functions would be evaluated in order to get comparable values of little bustard's niche breadth and position for the different site‐year niche spaces.

### 2.4 Statistical analysis

The effect of great bustard density on the degree of niche overlap between the species was analyzed using generalized linear mixed models (GLMMs) with Gaussian error distribution (*n* = 9 sites × year with sympatric occurrence of both species). Great bustard density for each study site and year was the number of all individuals observed, divided by the corresponding MCP area. We included study site as random factor in order to account for potential dependent effects between regions surveyed on several years.

We used GLMMs with Gaussian error distribution to evaluate shifts on little bustard niche comparing first situations of sympatry and allopatry (*n* = 26 sites × year). In order to test the hypothesis of niche release, we used niche breadth and niche position (two coordinates corresponding with each dimension of the two‐dimensional habitat niche) as the response variables, and the presence/absence of great bustard as the explanatory variable. We further analyzed whether intra‐ and interspecific density‐dependent effects caused niche variation, in order to evaluate the potential effects of density‐dependent competition using GLMMs. Niche breadth and position were used as response variables, and the explanatory variables were the density of little and great bustards inside the MCP. GLMMs testing potential density‐dependent competition only included those study sites and years where little and great bustards co‐occurred (*n* = 16 sites × year). In order to test for the functional response in habitat use, that is, the relative use depending on habitat availability (Mysterud & Ims, 1998), all models (allopatry/sympatry and density‐dependent) incorporated the niche breadth or position of the environmental niche as a covariate. Study site was also included as a random factor in all GLMMs in order to account for the potential dependency among data obtained in a given study region.

Observational bird data and land‐use maps were processed with ArcGis 9.3 (ESRI 2007). All statistical analyses and spatial calculations were performed with R software v3.1.1 (R Core Team, 2014). KDEs were built using the “ks” R package (Duong, 2014).

## 3 RESULTS

The lowest little bustard male density within the MCP was found in Camarma (0.50 males/km^2^) whereas Bellmunt showed the highest density (7.66 males/km^2^). Great bustard density varied more than little bustard density within the MCP from 0.38 birds/km^2^ in Calatrava South to 20.85 birds/km^2^ in Valdetorres (Table 1).

### 3.1 Habitat niche dimensions

The first three PCA habitat axes retained 80% of the variance. The first two PCA axes reflected a gradient of agrarian intensification, the first axis (PC1) being positively correlated with cereal cover, while the second axis (PC2) was positively correlated with the cover of ploughed fields but negatively correlated with the surface of young fallows, indicating a gradient of management of annual fallow (Table 2). The third axis (PC3) was mainly influenced by the cover of natural vegetation, and it can be interpreted as an index of semi‐permanent habitat availability (Table 2). These three PCA axes reflected the most important agrarian habitats used by the species during the breeding season. Therefore, we built three KDEs combining PC1‐PC2, PC1‐PC3, and PC2‐PC3 to evaluate niche overlap, breadth, and position in these three niche dimensions. One KDE for PC2‐PC3 habitat niche (the one obtained for Bellmunt site in 2008) was discarded for the analysis due to its odd shape.

**Table 2:** Results of the PCA to summarize original habitat variables. Only PCA axes considered as habitat niche dimensions are displayed

	PC1	PC2	PC3
Cereal	0.904	−0.013	−0.158
Young fallow	−0.280	−0.727	−0.443
Natural vegetation	−0.142	0.017	0.705
Ploughed field	−0.275	0.687	−0.494
Legume crop	−0.061	0.012	0.129
Dry woody culture	−0.053	0.017	0.124
Other	−0.043	−0.003	0.076
Explained variance (%)	48.3	18.1	13.6

### 3.2 Niche overlap between bustard species

The habitat niches of the little and the great bustard partially overlapped for the two‐dimensional niche spaces (mean for PC1‐PC2: 0.44; mean for PC1‐PC3: 0.42; mean for PC2‐PC3: 0.42. Values per study site can be found in Table S2). However, we found no effect of great bustard density on the degree of niche overlap for any two‐dimensional niche (PC1‐PC2: Estimate ± *SE* = 0.006 ± 0.005, *t* = 1.13, *p* = .34; PC1‐PC3: Estimate ± *SE* = 0.001 ± 0.003, *t* = 0.19, *p* = .86; PC2‐PC3: Estimate ± *SE* = 0.010 ± 0.008, *t* = 1.44, *p* = .25).

### 3.3 Shifts on little bustard niche breadth and position

Results show that habitat availability affected little bustard's niche, with niche breath increasing where the environmental niche was larger (Table 3). We also found a weak evidence of increased niche breadth in regions with great bustard presence for PC1‐PC3 habitat niche (Table 3; values per study site can be found in Appendix S1, Table S3). GLMMs for sympatric sites evaluating density‐dependent effects of intra‐ and interspecific competition on niche breadth showed that great bustard density was negatively related to little bustard niche breadth for PC1‐PC2 niche (Table 4). Likewise, we found a negative relationship between the density of little bustards and niche breadth of this species for the PC2‐PC3 niche (Table 4).

**Table 3:** Results of GLMMs analyzing the effects of great bustard presence on little bustard niche breadth and position controlling by the environmental niche (*n* = 26 for PC1‐PC2 and PC1‐PC3 analysis; *n* = 25 for PC2‐PC3 analysis). Two‐dimensional niches spaces were built using kernel density estimator and combinations of PCA axes (PC1‐PC2, PC1‐PC3, and PC2‐PC3) as habitat niche dimensions. Niche breadth was the number of cells of the two‐dimensional KDE falling within the 95% region, and niche position was estimated as the coordinates of each niche dimension where the two‐dimensional kernel density function attained the maximum probability value. All models included study site as random factor. Significant variables are highlighted in bold

2‐dimensional niche	Response variable	Explanatory variables	Estimates ± *SE*	χ^2^	*p*
PC1‐PC2	Breadth	Great bustard presence	427.282 ± 622.366	0.47	.492
		**Environmental niche breadth**	**0.664** ± **0.277**	**5.76**	**.016**
	Position dimension 1	Great bustard presence	−0.057 ± 0.169	0.11	.736
		**Position dimension 1 of environmental niche**	**0.556** ± **0.178**	**9.72**	**.002**
	Position dimension 2	Great bustard presence	0.080 ± 0.084	0.91	.342
		Position dimension 2 of environmental niche	0.095 ± 0.256	0.14	.712
PC1‐PC3	Breadth	Great bustard presence	681.470 ± 373.960	3.32	.068
		**Environmental niche breadth**	**0.615** ± **0.194**	**10.03**	**.002**
	Position dimension 1	Great bustard presence	−0.116 ± 0.155	0.56	.456
		**Position dimension 1 of environmental niche**	**0.628** ± **0.159**	**15.68**	<**.001**
	Position dimension 2	**Great bustard presence**	**0.312** ± **0.109**	**8.16**	**.004**
		Position dimension 2 of environmental niche	0.223 ± 0.195	1.30	.254
PC2‐PC3	Breadth	Great bustard presence	−107.761 ± 562.502	0.04	.848
		Environmental niche breadth	0.496 ± 0.345	2.07	.150
	Position dimension 1	Great bustard presence	0.070 ± 0.104	0.45	.501
		Position dimension 1 of environmental niche	0.062 ± 0.310	0.04	.843
	Position dimension 2	**Great bustard presence**	**0.385** ± **0.098**	**15.56**	<**.001**
		Position dimension 2 of environmental niche	0.253 ± 0.270	0.88	.350

**Table 4:** Results of GLMMs analyzing intra‐ and interspecific density‐dependent effects of competition on little bustard niche breadth and position controlling by the environmental niche. Two‐dimensional niches spaces were built using kernel density estimator and combinations of PCA axes (PC1‐PC2, PC1‐PC3, and PC2‐PC3) as habitat niche dimensions. Niche breadth was the number of cells of the two‐dimensional KDE falling within the 95% region, and niche position was estimated as the coordinates of each niche dimension where the two‐dimensional kernel density function attained the maximum probability value. Only sympatric sites were used in this analysis (*n* = 16 for each combination of PCA axes). All models included study site as random factor. Significant variables are highlighted in bold

2‐dimensional niche	Response variable	Explanatory variables	Estimates ± *SE*	*t*	*p*
PC1‐PC2	Breadth	**Great bustard density**	−**115.304** ± **42.224**	−**2.73**	**.029**
		Little bustard density	25.733 ± 201.063	0.13	.902
		Environmental niche breadth	−0.342 ± 0.360	−0.95	.373
	Position dimension 1	Great bustard density	−0.020 ± 0.014	−1.41	.202
		Little bustard density	0.081 ± 0.065	1.25	.250
		Position dimension 1 of environmental niche	0.237 ± 0.215	1.10	.307
	Position dimension 2	**Great bustard density**	−**0.042** ± **0.013**	−**3.29**	**.013**
		**Little bustard density**	−**0.098** ± **0.039**	−**2.49**	**.042**
		**Position dimension 2 of environmental niche**	**1.201** ± **0.364**	**3.30**	**.013**
PC1‐PC3	Breadth	Great bustard density	−5.412 ± 39.557	−0.14	.895
		Little bustard density	−51.247 ± 150.163	−0.34	.743
		Environmental niche breadth	0.349 ± 0.416	0.84	.430
	Position dimension 1	Great bustard density	−0.017 ± 0.015	−1.10	.308
		Little bustard density	0.066 ± 0.073	0.91	.394
		Position dimension 1 of environmental niche	0.235 ± 0.236	1.00	.353
	Position dimension 2	Great bustard density	0.001 ± 0.011	0.10	.924
		Little bustard density	−0.025 ± 0.047	−0.54	.609
		Position dimension 2 of environmental niche	0.195 ± 0.318	0.62	.558
PC2‐PC3	Breadth	Great bustard density	2.917 ± 36.095	0.08	.938
		**Little bustard density**	−**483.470** ± **179.238**	−**2.70**	**.031**
		Environmental niche breadth	−0.423 ± 0.412	−1.03	.339
	Position dimension 1	**Great bustard density**	−**0.042** ± **0.016**	−**2.70**	**.031**
		Little bustard density	−0.087 ± 0.049	−1.77	.121
		**Position dimension 1 of environmental niche**	**1.410** ± **0.450**	**3.14**	**.017**
	Position dimension 2	Great bustard density	−0.004 ± 0.011	−0.36	.729
		Little bustard density	−0.004 ± 0.041	−0.11	.918
		Position dimension 2 of environmental niche	0.069 ± 0.422	0.16	.875

Habitat availability also affected little bustard niche position for PC1 and PC2 dimensions, which were positively related to those of the environmental niche (Table 4). Little bustard niche position was also affected by the presence of great bustard for PC3 dimension, observed in both PC1‐PC3 and PC2‐PC3 niches (Table 3; values per study site can be found in Appendix S1, Table S4). We found that niche position was displaced toward higher values of PC3 under sympatry, indicating an increased use of natural vegetation in the presence of great bustard. The density of great and little bustards negatively influenced niche position for PC2 (Table 4). Intra‐ and interspecific competition induced a greater use of young fallows and decreased use of ploughed fields.

## 4 DISCUSSION

Our results based on the analysis of two‐dimensional habitat niches add empirical evidence to the role of intra‐ and interspecific competition in driving changes in species' ecological niches. They partially support previous evidences of interspecific competition between little and great bustards although some results depart from our initial hypotheses based on current ecological niche theory and deserve further investigation.

Habitats have long been considered as potential dimensions of species' ecological niches (e.g., Chase & Leibold, 2003; Schoener, 1989; Young, 2004). Because habitat selection is a fundamental mechanism mediating species coexistence (Morris, 2003; Rosenzweig, 1981), the study of habitat niches may bring novel insights on competition theory. Theories of habitat selection assume that interspecific competition causes a complete spatial separation of the species in their preferred habitats (Morris, 1988; Rosenzweig, 1981). As a consequence, one would expect no habitat niche overlap due to habitat niche divergence. However, the two‐dimensional habitat niches of these closely related species, the little and the great bustard, partially overlapped in those regions where they co‐occurred. This may be due to current unresolved interspecific competition, so that habitat niches are not yet completely segregated. Nonetheless, we did not find the theoretical expected negative relationship between niche overlap and the intensity of competition (May & Mac Arthur, 1972; Pianka, 1974). Our results are not the first to document this lack of relationship in currently competing species. Experiments conducted by Young (2004) found that asymmetric competition between two salmonid species did not cause reduced niche overlap because at high densities the habitat distribution of the competing species converged. This highlights the need to use additional measures of niche shift, other than the degree of niche overlap, to evaluate the existence and effects of interspecific competition.

When a species is released from a putative competitor, its niche breadth expands because interspecific competition no longer restricts the exploitation of resources previously monopolized by the competitor (Bolnick et al., 2010; Schoener, 1989). However, we found that little bustard niche breadth tended to increase in the presence of a competitor species for PC1‐PC3 (Table 3). Because we found a weak evidence, this result should be interpreted with caution and deserves further study in future. Two facts consistent with interspecific competitive processes may underlie this result: (1) a lower proportional use of a shared habitat and a higher use of the primary habitat, and (2) the incorporation of low‐quality habitats into little bustard males' habitat choice in order to reduce intraspecific competition within the species' primary habitat. Indeed, we found that little bustard niche position differed between allopatry and sympatry in the natural vegetation dimension (in both PC1‐PC3 and PC2‐PC3 niches; Table 3). Natural vegetation is one of the habitats most preferred by little bustard males (Delgado et al., 2010; Morales et al., 2005; Ponjoan, Bota, & Mañosa, 2012) and its proportional use was higher in sympatric than in allopatric conditions, in accordance with the density‐dependent change in little bustard habitat use found by Tarjuelo et al. (2017). Therefore, interspecific competition favors a shift in little bustard's habitat niche toward increased use of natural vegetation. In accordance with niche theory, the species assemblage seems to be governed by a “distinct habitat preference organization” because the little bustard increases the use of a primary habitat in the presence of a competitor (Morris, 1988).

Although theories of habitat selection state that coexisting species resolve their competition by complete segregation in different habitats (Morris, 1988; Rosenzweig, 1981), this is not always necessarily true. A species may still use a competitor's habitat even if the competitor is present in the community but its habitat choice is modified as a function of the competitor density, indicating that interspecific competition is operating (Morris, 2009). The proportional use of the common habitat decreases whereas those of other preferred habitats increase (Morris, 2009). Little and great bustards distribute in the same agrarian habitats when they live in sympatry and habitat exclusion is not apparent (Tarjuelo et al., 2017). When both species co‐occur, we found that the little bustard habitat niche breadth defined by PC1‐PC2 significantly decreased with great bustard density (Table 4), in agreement with ecological release theory (Schoener, 1989). Thus, little bustard males may reduce the proportional use of the habitat where they compete with great bustards (cereals, whose variation in the landscape is reflected by PC1) as interspecific competition intensifies. Competition with great bustard had also a density‐dependent negative effect on niche position for PC2 dimension, causing a higher use of young fallows as the density of great bustard increases (Table 4). This is in agreement with our hypothesis based on ecological niche theory and previous evidence of competition between both species (Tarjuelo et al., 2017). Competition with great bustard seems to induce density‐dependent variation in breadth and position of little bustard niche toward increased use of the primary habitat.

Contrary to theoretical predictions stating that intraspecific competition should expand the species niche because of a diversification of resource use (Svanbäck & Bolnick, 2007), we found that the density of little bustard was associated with a decrease in niche breadth for PC2‐PC3 niche in sympatry (Table 4). This contraction could be driven by variation in PC2 because niche position for this dimension was negatively influenced by little bustard density. This indicates a higher use of young fallows at high density of little bustards. This result could be related with the meaning of this PC axis, which represents a gradient of fallow‐ploughed field: while young fallow is a key habitat for little bustards, ploughed fields are barely used (Delgado et al., 2010; Morales et al., 2005). This limits the detection of resource diversification, which may likely occur in other habitat dimensions. We also acknowledge that the sample size used for mixed models of density‐dependent effects on little bustard habitat niche (*n* = 16) may be small to clearly separate the effects of intra‐ and interspecific competition. These results should encourage future studies that tease apart the relative importance of intra‐ and interspecific competition.

The little bustard habitat niche also depends on the particular landscape composition. More precisely, the availability of cereals and young fallows within the landscape affects little bustard niche breadth and position (Tables 3 and 4). The greater the share of these habitats in the landscape, the higher is their use by little bustards. Because landscape configuration modulates habitat selection (Morris, 2003), we recommend that ecological niche studies using habitats as resources to represent niche dimensions should control for the effects of habitat availability.

Our findings add new empirical evidence to the effects of competition on these bustard species. However, we acknowledge that this study has exclusively centered on the potential effects of competition on the habitat niche of little bustard males. Although results do not allow us to clarify whether great bustards also affect the habitat niche of little bustard females, this possibility should be borne in mind. Shared preferences between males and females of little bustard for particular habitats like fallows have been documented at landscape scale (e.g., Morales, Traba, Delgado, & García de la Morena, 2013; Tarjuelo et al., 2013) while habitat segregation seems to occur mainly at microhabitat scale (Morales et al., 2008). Therefore, future research is required to better understand the potential effects of interspecific competition on little bustard female's ecology, crucial for a declining species.

Under the current disappearance of nonproductive agrarian substrates and the recovery of the superior great bustard competitor (whose numbers have recently increased in many areas of Spain (Alonso & Palacín, 2010), attaining very high local densities (SEO/Birdlife, 2012)), the effects of competition on the habitat niche of the declining little bustard should be considered when designing conservation programs for the species. Future studies are required to evaluate the potential negative effects of interspecific competition with great bustard in little bustard's population dynamics. Likewise, interspecific competition may also induce changes on great bustard's ecological niche at finer scales, and future research is needed to elucidate this question (e.g., diet segregation Bonesi, Chanin, & Macdonald, 2004).

This is the first study addressing interspecific density‐dependent competition, habitat use, and species niche adjustments using a multidimensional niche method. Overall, our findings suggest that these bustard species are currently competing, perhaps induced by the recent changes in the dynamics of agricultural landscapes due to agricultural intensification. Most importantly, our study reveals density‐dependent effects of intra‐ and interspecific competition on a species' habitat niche, a fact that is still poorly understood. Our two‐dimensional habitat niche approach highlights relevant aspects of the quantification of species niche using kernel density estimators. The selection of niche dimensions is an important step in evaluating the role of interspecific competition in niche shifts and must rely on detailed knowledge of the species' ecological requirements. Empirical studies using computational tools which allow to easily obtain multidimensional niches should give more realistic insights on evolutionary and ecological processes shaping communities (Blonder et al., 2014). Studies of ecological niches aiming to improve our understanding of community organization require that intra‐ and interspecific competition are considered together, given their opposite effect on species' niches (Bolnick, 2001; Bolnick et al., 2010).

## CONFLICT OF INTEREST

None declared.

## AUTHORS CONTRIBUTION

R.T., M.M, and J.T conceived and designed the study and the statistical analysis. All authors contributed to fieldwork. R.T. analyzed the data and wrote the article. All authors reviewed the manuscript at different stages, and their wise comments greatly improved the scientific quality of the article.
