# Supplementary material for: Intraspecific and interspecific competition induces density‐dependent habitat niche shifts in an endangered steppe bird
Source: Ecol Evol. 2017 Oct 17;7(22):9720–30. doi: 10.1002/ece3.3444 (PMC5696386; doi:10.1002/ece3.3444)
Supplement: Supplementary file 1 [file ECE3-7-9720-s001.docx]

**Appendix S1**

Table A1. Proportion (percentage) of each agrarian habitat inside the minimum convex polygon (MCP) built with all little and great bustard observations for each study site and year. DWC (Dry woody cultures) include olive groves, vineyards and almond orchards. Other includes minor substrates like urban areas, or forests.

|  | Cereal | Young fallow | Natural vegetation | Ploughed field | Legumes | DWC | Others |
| --- | --- | --- | --- | --- | --- | --- | --- |
| Campo Real | 42.27 ± 3.09 | 9.87 ± 1.82 | 8.17 ± 0.64 | 18.28 ± 2.57 | 8.15 ± 4.30 | 5.59 ± 0.95 | 1.98 ± 0.24 |
| Valdetorres | 37.83 ± 16.81 | 21.28 ± 8.28 | 7.21 ± 0.83 | 26.48 ± 10.35 | 0.00 ± 0.00 | 0.00 ± 0.00 | 2.20 ± 1.30 |
| Daganzo | 20.23 | 27.14 | 9.24 | 31.58 | 0.00 | 0.00 ± 0.00 | 1.44 |
| Camarma | 25.24 | 10.51 | 11.53 | 22.03 | 1.64 | 0.36 | 10.87 |
| Calatrava North | 49.56 ± 6.95 | 13.30 ± 1.58 | 1.82 ± 2.01 | 17.78 ± 6.00 | 1.74 ± 1.79 | 6.86 ± 0.63 | 1.38 ± 0.50 |
| Calatrava South | 35.92 ± 8.43 | 16.46 ± 16.28 | 14.12 ± 4.86 | 7.93 ± 5.91 | 7.00 ± 8.94 | 5.35 ± 3.63 | 11.09 ± 2.11 |
| La Solana | 22.95 ± 4.42 | 8.73 ± 6.39 | 3.84 ± 0.92 | 13.36 ± 2.20 | 0.24 ± 0.34 | 37.52 ± 7.99 | 3.67 ± 2.01 |
| Bellmunt | 85.34 ± 1.22 | 1.98 ± 1.12 | 3.43 ± 0.20 | 1.55 ± 0.78 | 3.14 ± 2.10 | 0.26 ± 0.17 | 9.19 ± 13.81 |
| Belianes | 78.87 ± 3.69 | 2.67 ± 0.32 | 1.74 ± 0.35 | 1.59 ± 0.48 | 0.54 ± 0.51 | 9.93 ± 1.76 | 11.18 ± 15.99 |

Table A2. Mean (± SE) for the degree of niche overlap between the little and great bustards in each study site where the species co-occurred (n=9). Niche overlap was estimated as the volume under the area where a given pair of little and great bustard KDEs overlap. Zero values indicate no overlap whereas values of 1 reflect complete niche overlap.

| Site | Year | Overlap PC1-PC2 | Overlap PC1-PC3 | Overlap PC2-PC3 |
| --- | --- | --- | --- | --- |
| Campo Real | 2010-2012 | 0.58 ± 0.06 | 0.62 ± 0.03 | 0.56 ± 0.10 |
| Valdetorres | 2010-2011 | 0.63 ± 0.01 | 0.53 ± 0.02 | 0.61 ± 0.05 |
| Daganzo | 2010 | 0.35 | 0.37 | 0.37 |
| Camarma | 2006 | 0.42 | 0.42 | 0.28 |
| Calatrava North | 2008-2009 | 0.10 ± 0.­10 | 0.05 ± 0.06 | 0.10 ± 0.12 |

Table A3. Mean (± SE) for little bustard niche breadth in each study site (n=26 for PC1-PC2 and PC1-PC3, and n=25 for PC2-PC3). Niche breadth was measured as the as the number of cells of the two-dimensional KDE falling within the 95% probability defined region.

| Site | Year | Breadth PC1-PC2 | Breadth PC1-PC3 | Breadth PC2-PC3 |
| --- | --- | --- | --- | --- |
| Campo Real | 2010-2012 | 2425 ± 223 | 2321 ± 157 | 1293 ± 127 |
| Valdetorres | 2010-2011 | 4643 ± 1985 | 3531 ± 755 | 3345 ± 1747 |
| Daganzo | 2010 | 1949 | 4073 | 1462 |
| Camarma | 2006 | 2132 | 2172 | 3939 |
| Calatrava North | 2007-2011 | 3442 ± 543 | 1807 ± 676 | 1408 ± 644 |
| Calatrava South | 2007-2011 | 2239 ± 467 | 2851 ± 319 | 1532 ± 490 |
| La Solana | 2010-2011 | 3037 ± 1881 | 2170 ± 4 | 2262 ± 768 |
| Bellmunt | 2008-2011 | 330 ± 257 | 395 ± 199 | 165 ± 70 |
| Belianes | 2008, 2010-2011 | 757 ± 495 | 801 ± 552 | 292 ± 196 |

Table A4. Mean (± SE) for little bustard niche position in each study site (n=26 for PC1-PC2 and PC1-PC3, and n=25 for PC2-PC3). Niche position was estimated as the coordinates of each niche dimension where the two-dimensional kernel density function attained the maximum probability value.

| Site | Year | Niche PC1-PC2 | | Niche PC1-PC3 | | Niche PC2-PC3 | |
| --- | --- | --- | --- | --- | --- | --- | --- |
|  |  | Dimension 1 | Dimension 2 | Dimension 1 | Dimension 2 | Dimension 1 | Dimension 2 |
| Campo Real | 2010-2012 | 0.162 ± 0.237 | -0.004 ± 0.013 | 0.046 ± 0.293 | -0.015 ± 0.101 | -0.000 ± 0.013 | 0.046 ± 0.067 |
| Valdetorres | 2010-2011 | -0.401 ± 0.058 | 0.079 ± 0.032 | -0.483 ± 0.175 | -0.256 ± 0.093 | -0.039 ± 0.130 | -0.099 ± 0.047 |
| Daganzo | 2010 | -0.524 | 0.011 | -0.524 | 0.796 | 0.031 | 0.824 |
| Camarma | 2006 | -0.545 | 0.011 | -0.668 | -0.300 | -0.615 | -0.288 |
| Calatrava North | 2007-2011 | 0.094 ± 0.456 | -0.066 ± 0.351 | 0.056 ± 0.488 | -0.125 ± 0.121 | -0.039 ± 0.358 | -0.110 ± 0.124 |
| Calatrava South | 2007-2011 | -0.137 ± 0.537 | -0.139 ± 0.310 | -0.129 ± 0.531 | 0.029 ± 0.223 | -0.135 ± 0.320 | 0.019 ± 0.210 |
| La Solana | 2010-2011 | -0.483 ± 0.029 | -0.069 ± 0.241 | -0.596 ± 0.044 | -0.245 ± 0.078 | -0.154 ± 0.293 | -0.076 ± 0.142 |
| Bellmunt | 2008-2011 | 0.490 ± 0.010 | -0.012 ± 0.00 | 0.485 ± 0.00 | -0.015 ± 0.00 | -0.015 ± 0.000 | -0.021 ± 0.000 |
| Belianes | 2008, 2010-2011 | 0.457 ± 0.032 | -0.012 ± 0.00 | 0.450 ± 0.043 | -0.008 ± 0.013 | -0.015 ± 0.000 | -0.013 ± 0.013 |
